# A positive influence of basal ganglia iron concentration on implicit sequence learning

**DOI:** 10.1007/s00429-020-02032-7

**Published:** 2020-02-13

**Authors:** Jonas Persson, Benjamín Garzón, Rouslan Sitnikov, Lars Bäckman, Grégoria Kalpouzos

**Affiliations:** 1 Aging Research Center (ARC), Karolinska Institutet and Stockholm University, Tomtebodavägen 18A, 17165 Solna, Sweden; 2 School of Law, Psychology, and Social Work, Center for Developmental Research (CDR), Örebro University, Örebro, Sweden; 3 MRI Research Center, Karolinska University Hospital, Stockholm, Sweden

**Keywords:** Brain iron, fMRI, Sequence learning, Basal ganglia, Aging, Implicit, Caudate

## Abstract

Iron homeostasis is important for maintaining normal physiological brain functioning. In two independent samples, we investigate the link between iron concentration in the basal ganglia (BG) and implicit sequence learning (ISL). In Study 1, we used quantitative susceptibility mapping and task-related fMRI to examine associations among regional iron concentration measurements, brain activation, and ISL in younger and older adults. In Study 2, we examined the link between brain iron and ISL using a metric derived from fMRI in an age-homogenous sample of older adults. Three main findings were obtained. First, BG iron concentration was positively related to ISL in both studies. Second, ISL was robust for both younger and older adults, and performance-related activation was found in fronto-striatal regions across both age groups. Third, BG iron was positively linked to task-related BOLD signal in fronto-striatal regions. This is the first study investigating the relationship among brain iron accumulation, functional brain activation, and ISL, and the results suggest that higher brain iron concentration may be linked to better neurocognitive functioning in this particular task.

## Introduction

Implicit sequence learning (ISL) is a type of non-declarative memory that involves acquisition of complex motor skills. Such learning occurs when individuals demonstrate significant improvements in speed or accuracy of motor responses, but are typically unable to consciously recall what they have learned. ISL is typically studied using tasks in which a series of stimulus–response pairs forms a sequence that is either deterministic (Nissen and Bullemer 1987) or probabilistic (Howard and Howard 1997; Kóbor et al. 2018). Implicit learning is dependent on multiple learning processes, with differential contributions from statistical and sequence learning (Turk-Browne et al. 2010; Nemeth et al. 2013a, b; Kóbor et al. 2018). Statistical learning is the ability to extract regularities, or frequencies, from environmental input and may play an important role in language learning (Orbán et al. 2008; Turk-Browne et al. 2010), whereas sequence learning is defined as the acquisition of temporal order information in a series of stimuli (Nemeth et al. 2011). These may rely on different brain substrates (Rose et al. 2011; Kóbor et al. 2018; Simor et al. 2019), follow different learning curves (Simor et al. 2019), and are differentially affected by sleep (Doyon et al. 2009b).

In contrast to explicit learning, a number of studies have shown little or no impairment of ISL in aging (Howard and Howard 1997; Rieckmann et al. 2010). Studies also show that ISL is not differentially affected by sleep in older and younger adults (Nemeth et al. 2010), but there are observations showing less offline learning (i.e., consolidation) in older compared to younger adults when using a probabilistic second-order regularity sequence that enables separation between general skill learning and sequence-specific learning (Nemeth and Janacsek 2010). The general lack of age differences is somewhat surprising, as the basal ganglia (BG), which is a key region implicated in skill acquisition (Doyon et al. 2009a), exhibits marked age-related morphological and neurochemical losses (Bäckman et al. 2000; Raz et al. 2005; Walhovd et al. 2011).

Although previous reports have linked BG volume (Erickson et al. 2010) and activation (Poldrack et al. 2005) to procedural learning, less is known about the association between neurobehavioral underpinnings of motor skill acquisition and other biological events that occur with increasing age. Of particular interest in the current study is the relationship of BG iron concentration to neural and behavioral correlates of ISL.

Iron is critical for many biological functions in the brain, such as neurotransmitter synthesis, metabolism, and myelin synthesis (Ward et al. 2014). However, an overload of iron concentration may result in brain iron deposition that adversely impacts cellular functioning by causing neurotoxicity. Excessive brain iron deposition may lead to neurodegeneration via inflammation (Haider et al. 2014) and oxidative stress (Zecca et al. 2004; Mills et al. 2010).

Darki et al. (2016) recently reported a positive relationship between BG iron concentration and working memory in children and young adults, whereas a negative relationship between these variables is commonly observed in older adults (Penke et al. 2012; Daugherty et al. 2015; Kalpouzos 2018). Increased brain iron in the hippocampus has been linked to reduced word recall in older men (Bartzokis et al. 2011), and a longitudinal study recently demonstrated that higher BG iron concentration at baseline was linked to 2-year decline in working memory performance (Daugherty et al. 2015). In addition, higher BG iron concentration was associated with underrecruitment of fronto-striatal regions during a motor imagery task, and related to poorer recall of the imagined scenes in older adults (Kalpouzos et al. 2017). Currently, there is no consensus to what extent ISL is related to cognitive processes, including working memory. Some studies have demonstrated an association between working memory and implicit (e.g., Bo et al. 2011, 2012) and explicit (Christou et al. 2016) motor learning, while others have failed to find such a relationship (Janacsek and Nemeth 2013). Since both working memory and ISL implicate the basal ganglia, it is likely that they share some underlying process, and may both be affected by BG iron concentration.

A robust method to quantify brain iron concentration is quantitative susceptibility mapping (QSM; Deistung et al. 2013). This is a novel post-processing method that generates magnetic susceptibility maps that enable quantitative assessment of iron content in tissue composition. QSM is characterized by high sensitivity to local brain iron content, and thus provides an in-vivo estimate of brain iron (Deistung et al. 2013). Mapping of the relaxation rate $R_{2}^{ * }$ may also be used as a measure of iron concentration, and this link has been confirmed in post-mortem studies (Langkammer et al. 2010; Dusek et al. 2017). Recent work indicates that metrics derived from standard fMRI-EPI images are related to the signal intensity ratio (SIR), which has been used to estimate liver iron concentration (Gandon et al. 2004). SIR estimates are strongly correlated with $R_{2}^{ * }$ (Echeverría et al. 2012), and SIR-equivalent metrics from fMRI-EPI imaging could, therefore, be used for quantifying relative brain iron concentration in this region (Rombouts et al. 2007; Garzón et al. 2017).

The goal of the current study was to investigate associations among regional brain iron measurements, brain activation as assessed by functional MRI, and ISL in healthy adults. The use of an ISL task, the serial reaction time task (SRTT), was based on recurrent findings of brain iron accumulation in the BG (Hallgren and Sourander 1958; Bartzokis et al. 2007), and that these regions have been repeatedly implicated in sequence learning (Daselaar et al. 2003; Reiss et al. 2005; Rieckmann et al. 2010). In Study 1, younger and older adults (*n* = 36) were included to assess age effects on brain iron as measured with QSM and its links with performance and brain activity during the SRTT. In Study 2, we explored the link between fMRI-derived BG iron estimates, and ISL performance obtained offline in a large sample of older adults (*n* = 160) of similar age (64–68 years).

## Study 1

### Materials and methods

Study 1 aims at exploring the iron-ISL relationships at different ages using QSM, and relate these findings to ISL-dependent brain activity.

#### Participants

Forty-six individuals were recruited through newspaper advertisements and posted flyers. One younger and two older adults were excluded due to brain pathology, and two younger and two older adults were excluded because of technical scanner problems. QSM data were not collected for three additional participants. Thus, 21 younger adults and 15 older adults remained for analyses (Table 1). All participants were right-handed, did not report any previous or current neurological or psychiatric diseases, and no one was taking psychoactive medication. The Montreal Cognitive Assessment (MoCA) was used as a global screening tool for cognitive impairment (Nasreddine et al. 2005). We used a cut-off of 23, which corresponds to a score of 28 on the MMSE. Older adults had slightly lower educational level than younger adults (*P* = 0.05). The two age groups did not differ in vocabulary (*P* > 0.05). All procedures were approved by the Regional Ethical Review Board in Stockholm, and complied with the 1964 Helsinki declaration and its later amendments or comparable ethical standards. All participants signed informed consent prior to data collection.

**Table 1:** Study 1—participant characteristics and behavioral data

	Younger group	Older group
N (Women)	21 (12)	15 (7)
Age (Mean ± SD)	36.4 ± 4.6	69.7 ± 3.4^*^
Age range	26—42	65—77
Education^b^ (max = 3)	2.7 ± 0.4	2.3 ± 0.9
MoCA (max = 30)	28.1 ± 1.5	26.1 ± 1.9^*^
Vocabulary (max = 30)	24.5 ± 2.9	24.8 ± 4.9
Systolic blood pressure	115 ± 13	133 ± 16^*^
Diastolic blood pressure	75 ± 9	79 ± 10
Implicit sequence learning (SRTT)	mean (SD)	mean (SD)
Block 1–6 [RT^diff^]	15.3 (17.2)	24.9 (20.1)
Block 7–12 [RT^diff^]	24.7 (13.4)	32.2 (18.4)
Block 1–6 [errors^diff^]	0.7 (1.1)	2.4 (4.6)
Block 7–12 [errors^diff^]	0.5 (0.9)	0.5 (3.4)
Blocks 1–6 vs. 7–12 [RT^diff^]	–9.4 (15.6)	–7.3 (20.7)
Blocks 1–6 vs. 7–12 [errors^diff^]	–0.2 (1.5)	–1.9 (5.1)

For measures of blood pressure, an average of two independent measures of systolic and diastolic blood pressure was calculated

*MoCA* Montreal cognitive assessment, *SRTT* serial reaction time task, *ms* milliseconds, *SD* standard deviation

^*^Age group differences significant at *P* ≤ 0.05

^a^Educational level was assessed according to the highest degree obtained (1 = lower school certificate; 2 = high school, 3 = university). ^Diff^ = Difference between blocks of sequence and random as a measure of implicit learning (RT^diff^ = milliseconds; errors^diff^ = number of errors).

#### MRI acquisition for iron deposition and morphometry

Participants were scanned with an 8-channel phased array receiving head coil (Discovery MR750 3.0 T scanner, General Electric). T1-weighted 3D SPGR images were obtained with 0.94 × 0.94 × 1 mm^3^ voxel size (TR: 7.908 ms, TE: 3.06 ms, field of view: 24 cm, 176 axial slices, flip angle of 12°). A 3D multi-echo gradient-echo (MGRE) sequence was also acquired, with a spatial resolution of 0.94 × 0.94 × 1 mm^3^ TR: 37.52 ms, field of view: 24 cm, 146 axial slices, flip angle of 20°). The first echo time was equal to 3.74 ms and was followed by seven additional times, with a 3.752 ms interval between consecutive echoes.

#### Morphometry

Volumetric segmentation was performed with the Freesurfer 5.3 image analysis suite (https://surfer.nmr.mgh.harvard.edu/) on the T1 images (Fischl et al. 2002, 2004). The volume and susceptibility values of 6 regions were calculated from this segmentation: left and right caudate nucleus (CN), left and right globus pallidus (GP), and left and right putamen (PU). To obtain more robust estimates of QSM, the structure boundary was eroded and 15% of the most extreme values were removed prior to averaging.

#### Quantitative susceptibility mapping (QSM)

The frequency at each voxel was estimated from the complex multi-gradient-echo (MGRE) signal using non-linear least squares fitting (Liu et al. 2013) to obtain quantitative maps of susceptibility. Resulting frequency maps were subsequently spatially unwrapped with a 3D best-path unwrapping algorithm (Abdul-Rahman et al. 2009). Background inhomogeneities from low spatial frequency variations in the estimated frequency map stemming from air-tissue boundaries that produce background field gradients were removed using the regularized sophisticated harmonic artifact reduction software for phase data (RESHARP; Sun and Wilman 2014). This method applies Tikhonov regularization at the deconvolution stage of spherical mean-value filtering (Schweser et al. 2011). Subsequently, the non-linear variant (Liu et al. 2013) of morphology-enabled dipole inversion (Liu et al. 2012) was used to minimize discrepancies in the number of voxels belonging to edges between susceptibility and magnitude images. This method imposes a data-fidelity constraint determined by the difference between complex exponential functions of the observed and generated frequency maps. Details concerning the computation of these equations are reported in the previous citations. The MATLAB implementation of RESHARP and MEDI that were used are available at https://pre.weill.cornell.edu/mri/pages/qsm.html.

*Brain extraction, registration, and smoothing* A brain mask was obtained with the brain extraction tool (Smith 2002) from the FSL library (https://fsl.fmrib.ox.ac.uk). The root-mean square of the first four echoes of the MGRE sequence was used to obtain a rigid-body transformation to the T1-weighted image using the FSL tool FLIRT (Jenkinson et al. 2002). The T1-weighted images were subsequently nonlinearly registered to MNI space using the FNIRT tool (Andersson et al. 2007). The parameters obtained from these transformations were employed to project the maps of susceptibility onto each subject’s individual structural space and onto MNI space.

*Referencing QSM estimates* The QSM processing pipeline yields relative susceptibility values, which are not comparable across subjects due to the indetermination of the origin of k-space in the computation of susceptibility. To overcome this problem, and to select the most adequate reference site for our sample, we computed and inspected a map of standard deviation of $R_{2}^{ * }$, selecting the region with lowest variability as the center of the susceptibility reference region in MNI space. The selected reference region was located in the left corticospinal white-matter (WM) centered at [− 24; − 27; 39] (MNI coordinates) with a size of 1000 voxels. The rationale and details for this strategy are described in detail elsewhere (Garzón et al. 2017). An in-house python program was used for region creation, and the WM masks were obtained with the FSL segmentation algorithm FAST (Zhang et al. 2001).

*QSM analyses* Partial correlations were performed between QSM and (a) SRTT performance and (b) brain activation across all participants and within age groups, controlling for age, sex and education, as well as regional volume, as QSM and regional volumes were significantly correlated (see “Results”). Given that we did not have any regional hypotheses regarding the relationship of striatal iron to hippocampal and frontal activation, we used QSM estimates from the whole striatum in these analyses. For the association between striatal iron and striatal BOLD signal, we focused on the CN, as this region was specifically activated by the task. One outlier was removed in these analyses.

#### Serial reaction time task (SRTT)

In the SRTT, four squares were presented horizontally in the center of a computer screen. Each square position corresponded to one of four buttons, in order from left to right. Participants were instructed to press the corresponding buttons using the index and middle finger of each hand as quickly and accurately as possible when a white square turned gray. Response accuracy and reaction times (RT) were recorded with two MRI-compatible response boxes, one for each hand. Button presses were recorded using E-prime 2.0 (Psychology Software Tools, Inc., 2002). A blocked fMRI design was used, and the task was administered in two separate runs each including 12 blocks. Each block consisted of 36 trials, and each trial lasted 700 ms with a 300 ms inter-stimulus interval. In half of the blocks, and unknown to the participants, the trials followed a fixed second-order 12-item sequence with positions from left (1) to right (4) of 1–2–1–4–2–3–4–1–3–2-4–3 (Schendan et al. 2003). In the remaining blocks, new second-order random sequences were used and these sequences were never repeated. Sequence and random blocks were alternated, and each block was separated by a 17-s fixation period. Prior to scanning, participants performed a test run with 36 randomly presented stimuli to familiarize with the task. Error trials or omissions were excluded from analysis and median response times were used to minimize the influence of outliers.

#### Task fMRI acquisition and preprocessing

Functional data were acquired in a blocked fMRI design, using a gradient-echo-planar imaging (EPI) sequence (FOV = 22 cm, acquisition matrix 72 × 72 and slice thickness 3 mm—with additional zero-filling the matrix was filled to 128 × 128 with voxel size 1.7 × 1.7 × 3 mm^3^—flip angle 70°, TR = 2200 ms, TE = 30 ms, total accelerated (*R* = 2) EPI readout duration = 16.4 ms, 46 axial slices acquired in an interleaved bottom/up order). To allow for progressive saturation of the MR signal, 5 dummy scans were collected, and discarded prior to experimental image acquisition. The scanner task was presented on a projector, seen through a mirror mounted on the head coil.

All fMRI data were preprocessed using the statistical parametric mapping software (SPM12) implemented in MATLAB. Preprocessing comprised realignment and unwarp, slice timing correction to the first slice, and coregistration of the individual T1 image to the mean functional image. Following coregisteration, the T1 image was segmented into gray matter (GM) and WM, and the Diffeomorphic Anatomical Registration Through Exponentiated Lie Algebra (DARTEL) toolbox (Ashburner 2007) was used to create a custom group template from the segmented GM and WM images. In addition, deformations from the group-specific template to each of the subject-specific GM/WM images were computed (i.e., flow fields). Finally, the coregistered fMRI images were nonlinearly normalized, subject by subject, to the sample-specific template using the flow fields, affine aligned into the MNI template, and finally smoothed using an 8 mm FWHM Gaussian kernel. The final voxel size was 2 × 2 × 2 mm.

#### fMRI data analysis

Two runs of 270 volumes each were acquired for fMRI analyses. BOLD signal change between conditions was analyzed using the general linear model approach implemented in SPM12. A block-design matrix including all conditions of interest was specified using the canonical hemodynamic response function. In addition, six motion parameters were modeled as covariates. The onset of an epoch was set to the first stimulus in each condition. The resulting individual contrast images were submitted to a second-level analysis. The first and second half of the experiment were modeled separately (i.e., first half sequence, first half random, second half sequence, second half random). To estimate BOLD signal changes related to successful sequence learning, individual performance scores were used as regressors in the group analysis. Sequence learning (RT difference: sequence vs. random) was correlated with the corresponding BOLD signal difference between the sequence and random conditions in the second half using the multiple regression option in SPM12. Images were thresholded at *P* < 0.05 (FWE-corrected at cluster level; cluster-forming threshold at voxel-level *P* < 0.0001). A whole-brain analysis on the sequence > random contrast and sequence learning on blocks 4–6 as a regressor was conducted to examine BOLD-behavior relationships. These results reflect brain activation correlated with ISL on blocks 4–6. Note that we did not use ISL change as the dependent variable, because robust ISL was observed already in block 2.

Region-of-interest (ROI) analyses by means of extracting BOLD signal were performed using the Marsbar toolbox. Mean BOLD parameter estimate value was extracted from each condition for each participant. ROIs were functionally defined from the voxels that showed a significant relationship with sequence learning (see above). Each region was created by including significantly activated voxels within each functional cluster.

#### Experimental design and statistical analyses

*Associations among brain iron concentration, BOLD activation, brain volume, and ISL* First, partial correlations were performed to examine whether QSM was related to volumes of CN, GP, and PU. These partial correlations were performed across all participants as well as within each age group, controlling for age, sex and education. Second, relationships between QSM and ISL were assessed using partial correlations controlling for age, sex, education and striatal volume. Striatal volume was included as a covariate given its potential influence on ISL performance. Third, the association between brain iron and BOLD signal was determined using partial correlations between QSM and task-related ROIs that showed a positive relationship with ISL performance. These partial correlations were again performed in the whole group as well as within age groups, controlling for age, sex, education, and striatal volume. Note that the results were confirmed using $R_{2}^{ * }$ as an index of brain iron concentration, and that the results were generally consistent with the reported QSM findings. All statistical analyses were performed using SPSS. The interquartile range (IQR; quartile 3–quartile 1) rule of IQR × 3 was used for detecting the presence of outliers. The mediation analysis was conducted with the SPSS plugin PROCESS version 3 using the model 4 option and a simple mediation model with 1000 bootstrap samples and applied a confidence interval of 95% (percentile). CN iron concentration was used as a predictor, ISL as the outcome, and CN BOLD as the mediator. The associations were considered reliable if 95% CIs for the correlation coefficients did not include zero.

*Bootstrapping* Provided that the sample sizes were relatively small in Study 1, bootstrapping analyses were performed to test the reliability of the potential associations between QSM and ISL, as well as between QSM and task-related BOLD signal. These bootstrapping analyses were based on 1000 samples. The percentile confidence intervals (CIs) of parameter estimates for the correlation coefficients were used. The associations were considered reliable if 95% CIs for the correlation coefficients did not include zero.

### Results

#### Behavioral data show robust ISL in both younger and older adults

**Fig. 1:**
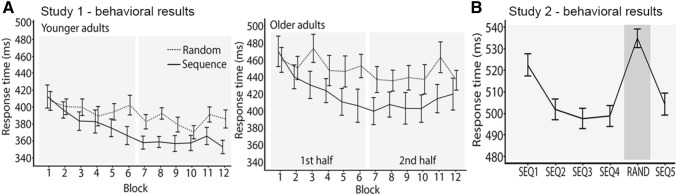
ISL performance in study 1 (**a**) and study 2 (**b**)

Accuracy was high, with a mean error rate of 4.2%. A 2 (condition: sequence vs. random) × 12 (blocks 1–12) × 2 (age group: younger vs. older adults) repeated-measures ANOVA on the RTs showed reliable main effects of condition (*F*(1,35) = 84.8, *P* < 0.001, $\eta_{{\text{p}}}^{2}$ = 0.73), block (*F*(11,35) = 14.9, *P* < 0.001, $\eta_{{\text{p}}}^{2}$ = 0.38) and age (*F*(1,35) = 11.1, *P* = 0.002, $\eta_{{\text{p}}}^{2}$ = 0.26). As expected, participants responded faster on sequence trials compared to random trials, and there was a general reduction in RT across blocks. Younger adults were generally faster than older adults. Moreover, the non-significant age × block (*F*(1,35) = 0.74, *P* = 0.69, $\eta_{{\text{p}}}^{2}$ = 0.023) and age × block × condition (*F*(1,35) = 1.41, *P* = 0.17, $\eta_{{\text{p}}}^{2}$ = 0.04) interactions indicated similar learning rates in both age groups. Importantly, the block × condition interaction was significant (*F*(11,35) = 5.92, *P* < 0.001, $\eta_{{\text{p}}}^{2}$ = 0.16) reflecting that RTs for the sequence condition were differentially reduced compared to the random condition across blocks (Fig. 1, Table 1).

#### QSM results demonstrate higher BG iron content in older compared to younger adults

The iron concentrations, calculated across age groups, showed regional variability in line with previous findings (Betts et al. 2016). A 2 (age group) × 4 (region) ANOVA showed main effects of region (*F*(1,35) = 547.5, *P* < 0.001, $\eta_{{\text{p}}}^{2}$ = 0.95), and age (*F*(1,35) = 31.8, *P* < 0.001, $\eta_{{\text{p}}}^{2}$ = 0.33), and a reliable age by region interaction (*F*(1,35) = 8.94, *P* < 0.001, $\eta_{{\text{p}}}^{2}$ = 0.48). Despite this interaction, older adults had higher iron concentrations in all regions compared with younger adults (CN: *F*(1,35) = 15.3, *P* < 0.001, $\eta_{{\text{p}}}^{2}$ = 0.28; PU: (*F*(1,35) = 29.5, *P* < 0.001, $\eta_{{\text{p}}}^{2}$ = 0.43); GP (*F*(1,35) = 8.21, *P* = 0.007, $\eta_{{\text{p}}}^{2}$ = 0.17).

#### A whole-brain analysis showed that ISL was linked to increased brain activation in fronto-striatal regions, thalamus and hippocampus

**Fig. 2:**
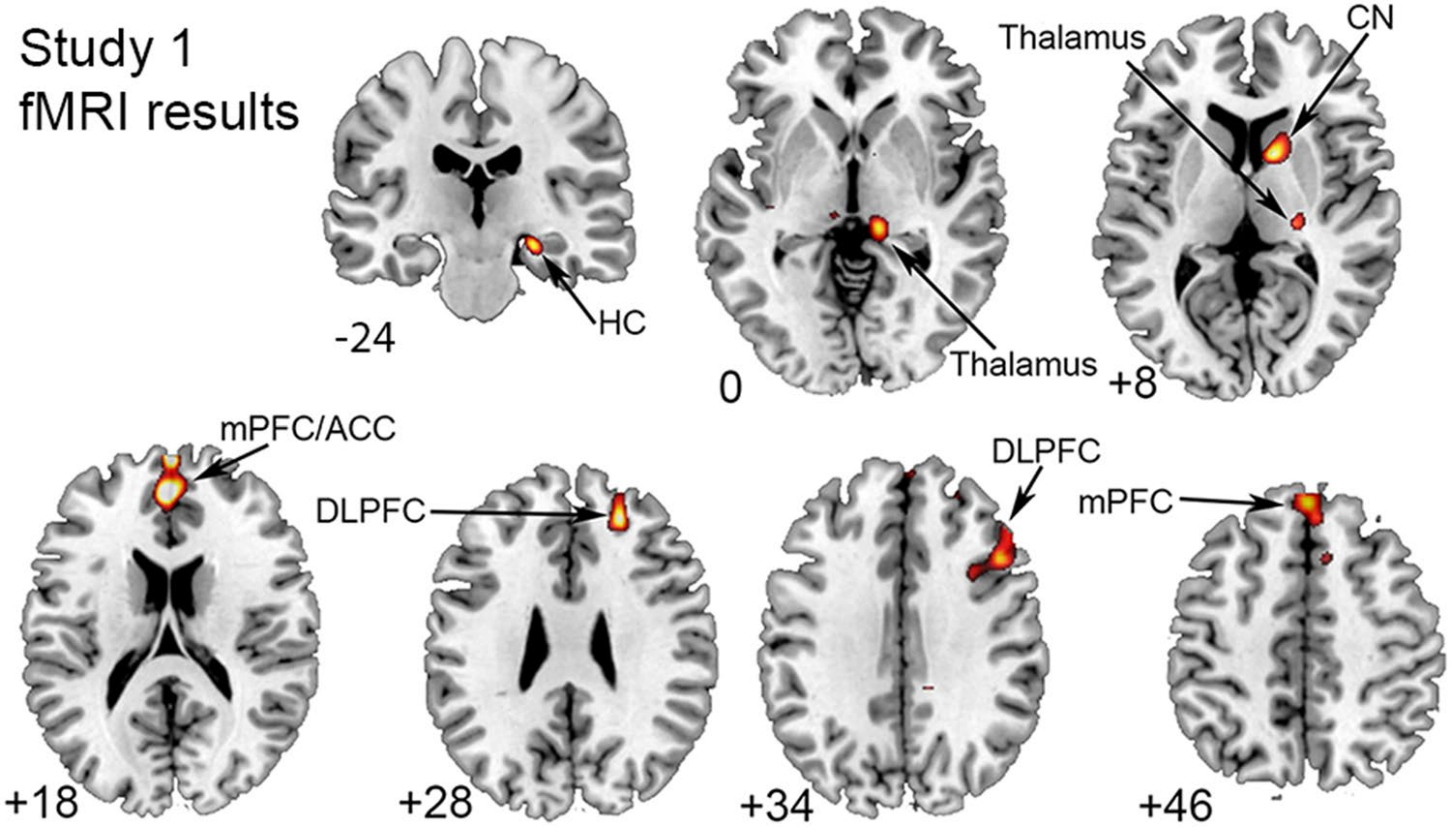
Brain activation associated with implicit sequence learning. Results are displayed at a cluster corrected threshold of *p*FWE < 0.05. All results are reported in MNI space. Activation is shown on transverse sections of the brain, except for the hippocampus (top) which is shown on a coronal section of the brain

Across all participants, ISL was significantly related to activation in striatal and medial and lateral frontal regions, as well as in thalamus and hippocampus (Fig. 2). No significant activation decreases associated with sequence learning were found. A follow-up ROI analysis performed on the significant clusters from the whole-brain analysis showed non-significant effects of age (all *P*s > 0.1).

#### Volumetric data

Regional volumes were adjusted for total intracranial volume (TIV) using the formula, Volume_adji_ = Volume_rawi_ – *b*(TIV_i_ – TIV_mean_), where *b* is the slope of volume regression on TIV (Jack et al. 1989). A 2 (age group) × 3 (region) ANOVA showed a main effect of region (*F*(1,35) = 1224.2, *P* < 0.001, $\eta_{{\text{p}}}^{2}$= 0.97), age (*F*(1,35) = 15.1, *P* < 0.001, $\eta_{{\text{p}}}^{2}$ = 0.31), and a significant age by region interaction (*F*(1,35) = 21.1, *P* < 0.001, $\eta_{{\text{p}}}^{2}$ = 0.38). Older adults had overall smaller volumes compared to younger adults, with the largest age effects in PU (*F*(1,35) = 35.5, *P* < 0.001, $\eta_{{\text{p}}}^{2}$ = 0.46), and a marginally significant effect in GP (*F*(1,35) = 3.85, *P* = 0.057, $\eta_{{\text{p}}}^{2}$ = 0.09). The age difference in the CD was non-significant (*F*(1,35) = 1.42, *P* = 0.24, $\eta_{{\text{p}}}^{2}$ = 0.03). Partial correlations with age as a covariate revealed significant relationships of ISL to striatal (*r* = 0.326, *P* = 0.04) and GP (*r* = 0.344, *P* = 0.029) volumes, indicating that larger volumes were related to better learning.

#### QSM–volume relationships

No reliable correlation was found between iron concentration and volume in any of the ROIs examined, neither when all participants were included (all *P*’s > 0.05), nor in the age groups tested separately (all *P*’s > 0.05).

#### Higher brain iron content was associated with better ISL

**Fig. 3:**
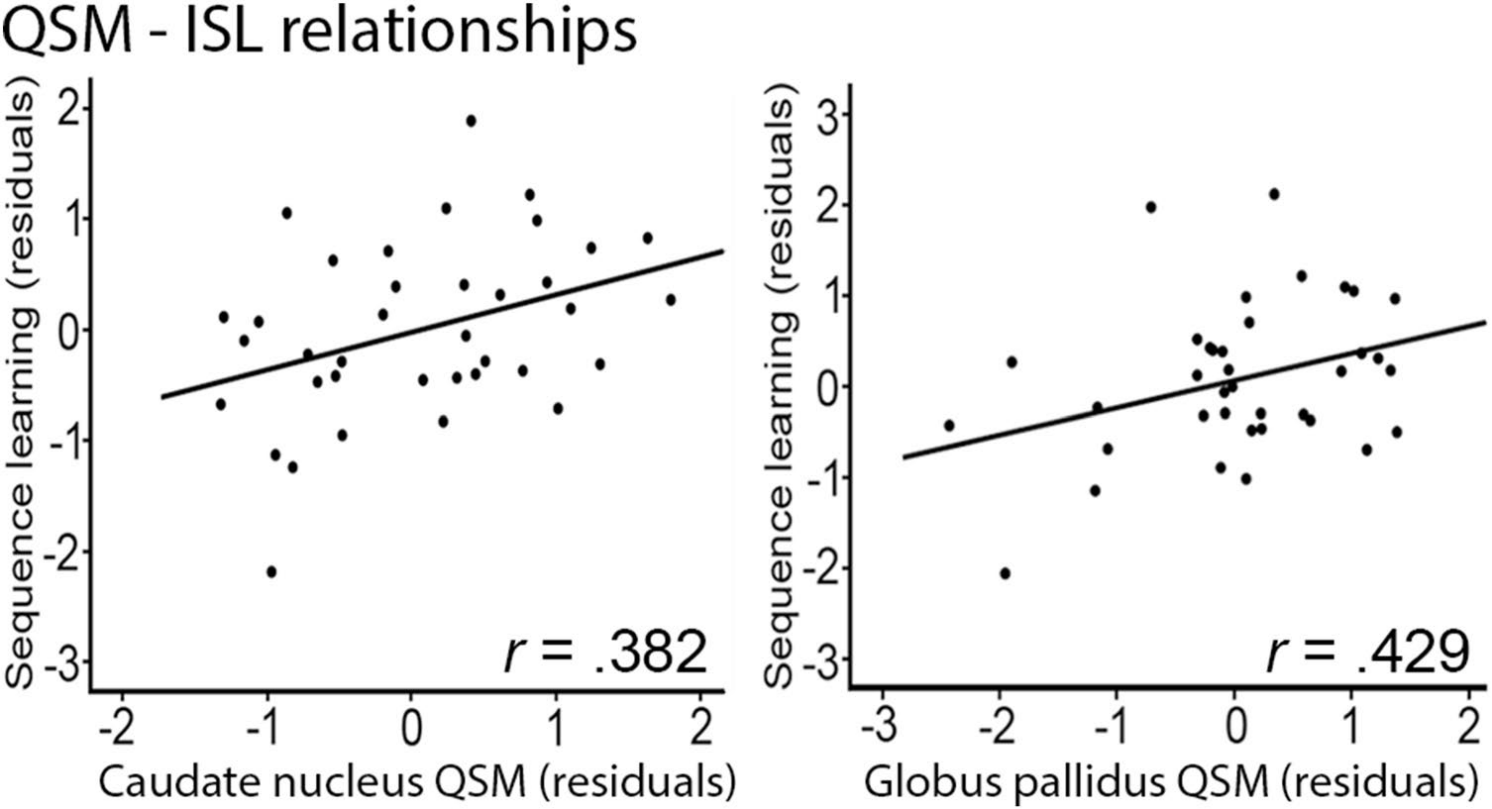
Relationships of striatal and GP iron concentration to ISL. Correlations (all participants) between QSM and SL [residuals adjusted for age and CN (**a**), GP (**b**) and striatal (**c**) volume]. *GP* globus pallidus, *CN* caudate nucleus

Across all participants, significant positive iron—ISL correlations were found for CN, and GP (Fig. 3; CN: *r* = 0.382, *P* = 0.026, 95% bootstrap CI: 0.002 to 0.705; GP: *r* = 0.429, *P* = 0.011, 95% bootstrap CI 0.065–0.677), but not for PU (*P* > 0.05, 95% bootstrap CI − 0.191 to 0.538). Within age groups, no significant relationships between QSM and ISL were found (all *P*’s > 0.05).

#### More brain iron was related to increased brain activation

**Fig. 4:**
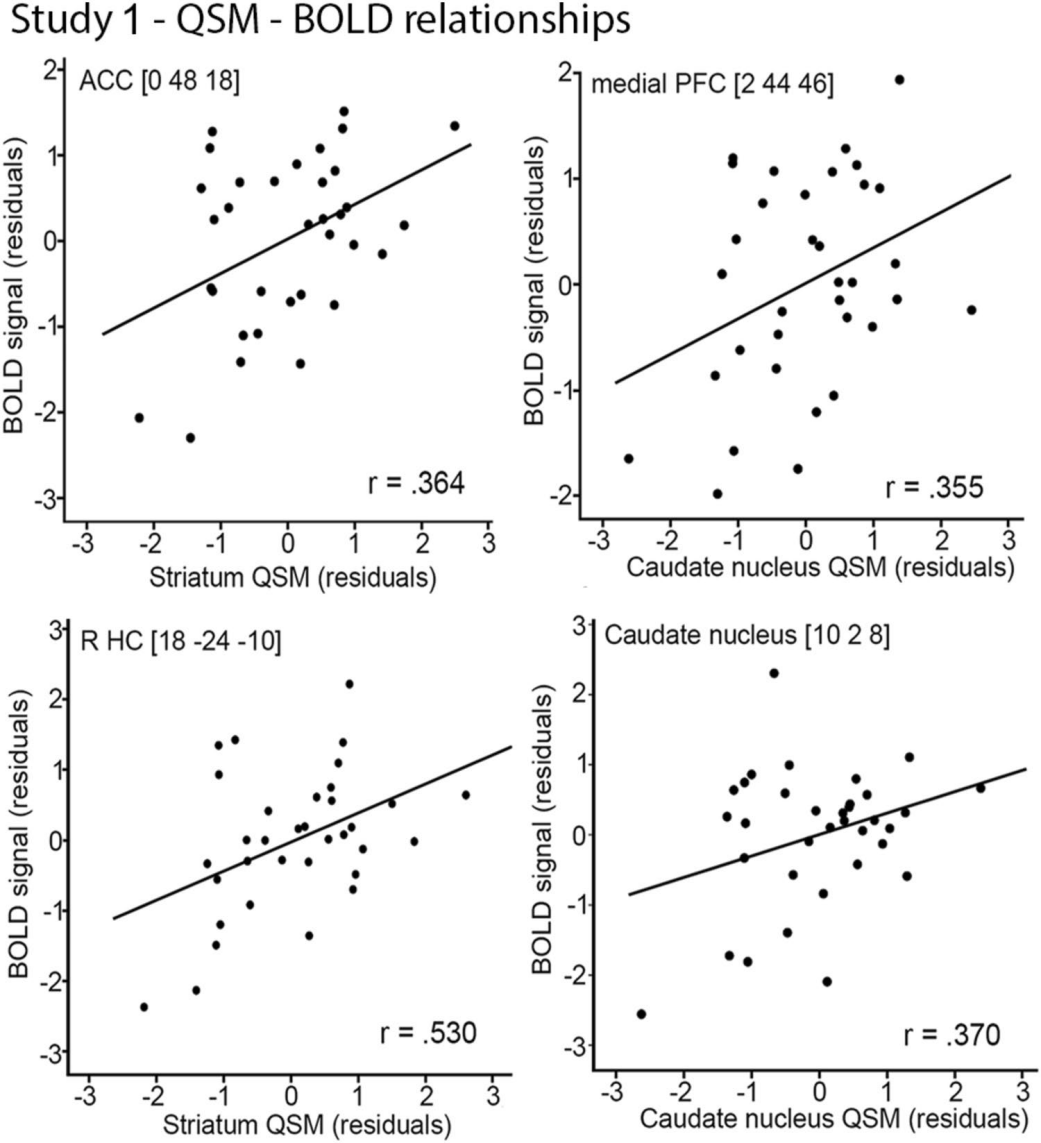
Relationships of striatal and GP iron concentration to task-related brain activation. Correlations (all participants) between QSM and BOLD signal (residuals adjusted for age and regional volume

Across all participants, striatal QSM estimates were positively associated with BOLD signal in anterior cingulate cortex (ACC; *r* = 0.364, *P* = 0.041), right hippocampus (*r* = 0.530, P = 0.002), and medial frontal cortex (*r* = 0.355, *P* = 0.046). These associations showed that more striatal iron was related to increased activation in these regions (Fig. 4). Moreover, iron concentration was positively related to BOLD signal in right CN (*r* = 0.37, *P* = 0.037). No significant correlations were found within age groups (all *P*’s > 0.05).

#### The mediation analysis showed that the relationship between brain iron and ISL was mediated by caudate BOLD activation

As a first step, we established that all zero-order correlations in this model were significant (*P* < 0.05). The mediation analysis included ISL as the dependent variable, CN iron concentration as the independent variable, and CN BOLD as the mediator. This analysis showed that the effect of CN iron concentration on ISL was no longer significant when CN BOLD activation was included as a mediator indicating that the CN iron concentration—ISL association was mediated by CN BOLD activation. A nonparametric bootstrapping analysis using a 95% bias-corrected bootstrap confidence interval (CI) for the indirect effect was above zero (CI upper level: 0.0001; CI lower level: 0.0012) indicating significant mediation.

## Study 2

### Materials and methods

Because the results of Study 1 were at variance with previous findings in older adults in general, but also particularly with this same sample of participants (Kalpouzos et al. 2017; Salami et al. 2018), where higher brain iron was related to poorer motor and cognitive performance, a second study was conducted to confirm the findings from Study 1. Design, recruitment procedure, imaging protocols and details of the cognitive and lifestyle battery for Study 2 has been described previously (Nevalainen et al. 2015).

#### Participants

One-hundred and eighty older (64–68 years; mean 66.2; SD 1.2; 81 women) participants, randomly selected from the population register of Umeå, were included in the initial sample. Exclusion criteria were pathological deviations in brain and cognitive functions or circumstances that may bias task performance or obstruct brain imaging (e.g., metal implants). Seventeen participants were excluded based on poor quality T1 images. Three additional participants were excluded (pathological findings: 2; outlier: 1). Thus, the final sample consisted of 160 participants. Demographic and medical information, along with ISL performance is provided in Table 2. The Mini-Mental State Examination (MMSE; Folstein et al. 1975) was used to screen for global cognitive disturbances. Only participants with a score of 27 or higher were allowed into the study. All procedures performed in the study were approved by the Regional Ethical Review Board in Umeå, and complied with the 1964 Helsinki declaration and its later amendments or comparable ethical standards. All participants provided signed written informed consent prior to testing.

**Table 2:** Study 2—participant characteristics and behavioral data

N (Men/Women)	160 (87/74)
Age (SD)	66.2 (1.2)
Age range	64–68
Education^b^ (SD)	13.5 (3.5)
MMSE (SD, range)	29.3 (0.8, 27–30)
Vocabulary (SD; max = 30)	23 (4.1)
Systolic blood pressure (SD)	142 (17.6)
Diastolic blood pressure (SD)	85 (9.8)
Implicit sequence learning (SRTT)	ms (SD)
Block 1	522 (65)
Block 2	501 (61)
Block 3	497 (60)
Block 4	498 (62)
Block 5	534 (54)
Block 6	504 (65)
MotDiff	33 (33)

*MMSE* mini-mental state examination, *MotDiff* block 5 − block 4 + block 6/2, *SRTT* serial reaction time task, *ms* milliseconds, *SD* standard deviation

^a^Educational attainment was assessed by number of years in school (< 10 years: elementary school; 10–13 years: high school; > 13 years: college)

#### MRI acquisition for morphometry and iron deposition

Participants were scanned with a 32-channel phased array receiving head coil (Discovery MR750 3.0 T scanner, General Electric). T1-weighted 3D SPGR images were obtained (TR: 8.2 ms, TE: 3.2 ms, field of view: 25 cm, 176 axial slices, flip angle of 12°). Functional MRI data were acquired at rest using a T2*-weighted single-shot gradient-echo-planar imaging sequence (FOV = 25 cm, acquisition matrix 96 × 96 and slice thickness 3.4 mm, 0.5 mm spacing, flip angle 80°, TR = 2000 ms, TE = 30 ms, 37 axial slices acquired), and used to estimate local brain iron content. A total of 170 volumes were collected. To allow for progressive saturation of the MR signal, 10 dummy scans were collected, and discarded prior to experimental image acquisition.

#### Preprocessing

Functional data were preprocessed using the Data Processing Assistant for Resting-State fMRI (Chao-Gan and Yu-Feng 2010), and has been described in detail elsewhere (Nyberg et al. 2016). First, correction for acquisition time differences between slices within each volume, and motion correction was performed. A within-subject rigid registration was carried out to align functional and structural T1-weighted images. Second, removing of effect of physiological noise was performed by regressing out Friston’s 24 parameters from a motion model as well as nuisance variables (i.e., global signal, WM, and cerebrospinal fluid) along with both linear and quadratic trends. In addition, nuisance-corrected data were bandpass filtered (passband 0.01–0.1 Hz). Finally, the noise-corrected realigned fMRI images were nonlinearly normalized to the sample-specific group template using DARTEL (Ashburner, 2007), and subsequently affine aligned into stereotactic Montreal Neurological Institute (MNI) space. In the last step, images were smoothed using a 6 mm FWHM Gaussian filter.

#### Estimation of iron concentration and morphometry

For each participant, the previously preprocessed resting-state fMRI volumes were averaged. We referenced susceptibility values with respect to the average in a region in the corticospinal tract (MNI coordinates [− 26; − 26; 34]; see rationale under 2.1.3 above), which is among the fiber bundles most resilient to age-related degeneration (de Groot et al. 2014). Metrics (here denoted *V*_EPI_) from the averaged T2* functional images were used to estimate iron concentration (similar to the method described in Garzón et al. 2017). This measure has been associated with transverse relaxation rates ($R_{2}^{*}$), and can be used for in-vivo iron quantification (Langkammer et al. 2010). A similar estimation was performed for the WM reference region. Finally, a relative measure [Δ*V*_EPI_ = *V*_EPI_ – *V*_EPI_.ref] was computed. T1-weighted MR images were segmented into GM, WM, and cerebrospinal fluid using the unified segmentation approach (Ashburner and Friston 2005), and further spatially normalized using DARTEL and smoothed in SPM12 (Statistical Parametric Mapping, Wellcome Trust Centre for Neuroimaging, https://www.fil.ion.ucl.ac.uk/spm/) implemented in Matlab (The Mathworks, Inc). Regions of interest were defined using the automatic anatomical labelling system (AAL) implemented in the WFU_Pickatlas, and the means of both GM volume and *V*_EPI_ images were extracted for each participant using Marsbar (https://marsbar.sourceforge.net/) for the following regions: GP, CN, PU, and the striatum (CN + PU).

#### Morphometry

The “light cleanup” option was used to remove odd voxels from the segments. The grey-matter images were further analyzed using DARTEL (Ashburner 2007) in SPM12b. The grey-matter segments were imported into DARTEL space, and a customized grey-matter template was created including subject-specific flow fields containing the individual spatial normalisation parameters (diffeomorphic non-linear image registration). By incorporating the affine transformation of the DARTEL template to Montreal Neurological Institute (MNI) space, the grey-matter segments were further warped into standard MNI space. To preserve local-tissue volumes, the normalized grey-matter volumes were modulated by scaling them with Jacobian determinants from the registration step. Volumes were smoothed with a full-width at half-maximum Gaussian kernel of 8 mm in three directions.

#### SRTT

The task procedure for SRTT was similar to that used in Study 1 with a few exceptions. First, the experiment was performed offline and consisted of 6 blocks, each containing 48 items. In block 1–4 and 6, unknown to the participants, the trials followed a fixed second-order 12-item sequence with positions from left (1) to right (4) of 1–2–1–4–2–3–4–1–3–2–4–3 that were repeated 4 times, whereas block 5 consisted of 4 repetitions of new second-order 12-item sequences. Second, each trial lasted 750 ms with a 250 ms inter-stimulus interval (ISI) and the practice session prior to testing consisted of 2 blocks with 24 items each. Third, the primary SRTT outcome measure in Study 2 was the difference in RT between random and repeated sequences (block 5 − block 4 + block 6/2). In all behavioral analyses of ISL, error trials or omissions were excluded. Partial correlations, controlling for age, sex, and education were computed between iron estimates and ISL. As the results showed significant relationships between regional volume and brain iron, volume was also controlled for.

#### Experimental design and statistical analyses

Relationship between brain iron concentration, brain volume, and ISL. First, partial correlations were performed to examine whether *V*_EPI_ was related to volumes of CN, GP, PU, as well as the whole striatum (CN and PU). These partial correlations were performed across all participants controlling for age. Second, relationships between iron concentration and ISL were assessed using partial correlations controlling for age and local volume. Local volume was included as a covariate as it may be a confounding factor given its potential influence on ISL. All statistical analyses were performed using SPSS. The interquartile range (IQR; quartile 3–quartile 1) rule of IQR × 3 was used for detecting the presence of outliers.

### Results

#### Iron concentration was negatively related to striatal volume

Partial correlations with age as a covariate showed significant negative correlations between iron concentration and striatal volume across all included regions, except GP. Correlations between brain volume and ISL were all non-significant (all *P*’s > 0.05).

#### Behavioral data show robust ISL in the age-homogenous group of older adults

A repeated-measures ANOVA showed a reliable difference between block 5 (random) and the average of blocks 4 and 6 (sequence; *F*(1,159) = 165.5, *P* < 0.001, $\eta_{{\text{p}}}^{2}$ = 0.51), reflecting that RTs for the sequence blocks were shorter than for the random blocks thus indicating robust ISL (Fig. 1,Table 2).

#### As in Study 1, iron concentration was positively related to ISL

**Fig. 5:**
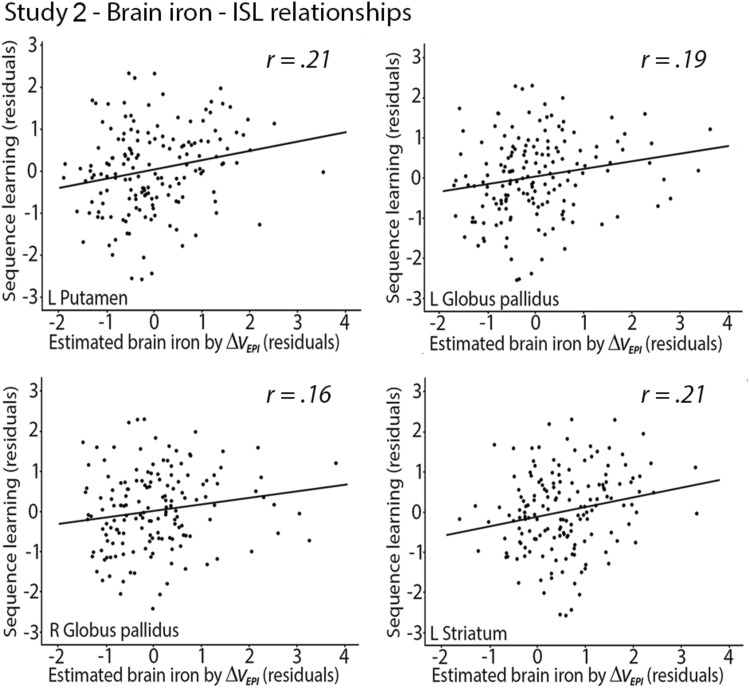
Relationships of putamen, globus pallidus, and striatal iron concentration to ISL. Correlations between Δ*V*_EPI_ and ISL (residuals adjusted for age and local BG volume)

Brain volume correlated negatively with brain iron in many regions, and these results are reported in Table 3. Partial correlations between brain iron concentration and ISL were positive and significant in left PU (*r* = 0.212, *P* = 0.007), left GP (*r* = 0.19, *P* = 0.017), right GP (*r* = 0.163, *P* = 0.04), and left striatum (*r* = 0.208, *P* = 0.008), indicating that higher BG iron concentration was related to better ISL performance (Fig. 5).

**Table 3:** Relationships between brain iron concentration and brain volumes

	Estimated brain iron with Δ*v*_EPI_							
	L CN	R CN	L GP	R GP	L PU	R PU	L striatum	R striatum
Brain volume								
L CN	− 0.230^**^	− 0.213^*^	− 0.054	− 0.043	− 0.200^*^	− 0.146	− 0.241^**^	− 0.190^*^
R CN	− 0.213^**^	− 0.235^**^	− 0.065	− 0.056	− 0.194^*^	− 0.156^*^	− 0.228^**^	− 0.205^**^
L GP	− 0.161^*^	− 0.158^*^	0.153	0.136	− 0.137	− 0.123	− 0.166^*^	− 0.149
R GP	− 0.206^**^	− 0.194^*^	0.140	0.142	− 0.141	− 0.123^**^	− 0.193^*^	− 0.167^*^
L Putamen	− 0.312^***^	− 0.252^**^	0.144	0.130	− 0.258^**^	− 0.171^*^	− 0.322^***^	− 0.224^**^
R Putamen	− 0.310^***^	− 0.298^***^	0.121	0.127	− 0.243^**^	− 0.215^**^	− 0.313^***^	− 0.273^***^
L Striatum	− 0.292^***^	− 0.248^**^	− 0.048	− 0.012	− 0.246^**^	− 0.168^*^	− 0.303^***^	− 0.220^**^
R Striatum	− 0.288^***^	− 0.290^***^	− 0.057	− 0.031	− 0.238^**^	− 0.203^**^	− 0.296^***^	− 0.261^**^

*CN* caudate nucleus, *GP* globus pallidus, *PU* putamen

^*^ < 0.05; ** < 0.01; *** < 0.001;

## Discussion

The present investigation examined the potential link between brain iron concentration and neurocognitive markers of ISL. The results from Study 1 showed that younger and older adults demonstrated significant and comparable ISL, and performance gains were related to activation in a fronto-striatal brain network. A major result of Study 1 was that more striatal iron was linked to increased BOLD signal in these regions, regardless of age and local regional volume. Moreover, findings from Study 1 indicate that higher BG iron concentration was related to better ISL, and the results from Study 2 confirmed the positive link between brain iron concentration and ISL, using a large age-homogenous sample. Thus, converging evidence from two independent studies suggests a positive association between brain iron and ISL.

The results from Study 1 demonstrate that both younger and older adults showed robust ISL of similar magnitude. The sizeable inter-individual differences in ISL, along with the large ISL effect are in good agreement with previous findings (Rauch et al. 1997; Peigneux et al. 2000; Reiss et al. 2005; Rieckmann et al. 2010). Although a lack of age differences in sequence learning is not uniformly demonstrated (Curran 1997; Howard et al. 2004), the present results concur with the bulk of past research reporting small or no age differences in ISL (Cherry and Stadler 1995; Howard and Howard 1997; Daselaar et al. 2003; Aizenstein et al. 2006; Rieckmann et al. 2010). Concerning age comparisons in brain activation associated with ISL, our results largely corroborate previous findings of small or non-existent differences in striatal activation between younger and older adults (Daselaar et al. 2003; Rieckmann et al. 2010).

Across younger and older adults, we found activation of a functional brain network that included the CN, thalamus, PU, ACC, and hippocampus, along with lateral and medial frontal cortex to be involved in ISL. Importantly, BG iron concentration was positively associated with brain activation in several of these regions, including ACC, medial frontal cortex, hippocampus, and CN. Thus, BG iron concentration affected both ISL and brain activation critical for efficient ISL. Both animal and human work support the role of fronto-striatal circuits in motor skill learning (Jenkins et al. 1994; Doyon et al. 2009a; Yin et al. 2009), and our results confirm past work implicating that a broad network of regions supports sequence-specific learning. It is well-known that BG plays an essential role in planning, learning, and execution of implicit motor skills (Doyon et al. 2009a). Brain imaging evidence demonstrates increased striatal activation during ISL (Rauch et al. 1997; Reithler et al. 2010; Rieckmann et al. 2010; Debas et al. 2014). We also found that hippocampus activation was correlated with ISL. Although hippocampus is most commonly associated with explicit memory, it may play a role also during implicit learning, possibly in interaction with BG (Albouy et al. 2008). Thalamus may support ISL by serving as a hub for integrating visuospatial input important for perception and attention with regions devoted to action control, and lateral and medial frontal cortex likely subserve communication between BG and the medial temporal lobe (Poldrack and Rodriguez 2004; Seger and Cincotta 2006).

Although the finding of a positive link between brain iron and ISL across both younger and older adults in Study 1 was somewhat unexpected, the results from Study 2 largely support the positive relationship between brain iron concentration and sequence learning in a relatively large sample of older adults. It should be noted that we used QSM to estimate brain iron concentration in Study 1. Given its higher sensitivity to local susceptibility, QSM may provide a more accurate estimate of brain iron compared to metrics derived from functional images as used in Study 2. However, although the metrics used to estimate brain iron concentration in Study 2 may be less reliable, we were still able to replicate the findings from Study 1 of a positive relationship between BG iron concentration and ISL. In both studies, these findings were independent of age and BG volume. The finding that similar links between brain iron and ISL were observed in these two studies using different ways of assessing iron and different version of the SRTT should strengthen the generalizability of the findings.

Although most studies to date have found a negative relationship between brain iron and cognition in older adults (Rodrigue et al. 2013; Kalpouzos et al. 2017), this link is inversed in younger populations. A common finding in studies involving children (Darki et al. 2016) and young adults (Allen et al. 2017) is that higher brain iron concentration is related to better cognitive performance.

At first glance, the current results are difficult to reconcile with our recent finding that striatal brain iron level was negatively related to fronto-striatal activation in a motor imagery task, and to explicit memory performance in older adults (Kalpouzos et al. 2017; Salami et al. 2018). However, the current data and those reported by Kalpouzos et al. were derived using two completely different tasks that rely on distinct memory systems. These results, and recent observations that brain iron concentration was linked to visual but not verbal memory (Darnai et al. 2017), suggests that the behavioral effects of brain iron may be task-dependent. Another possibility is that brain iron may modify the balance between memory systems in a way that facilitates automatic acquisition of motor skills. It should also be emphasized that the negative brain iron—behavior link demonstrated in previous studies primarily used accuracy as the outcome measure, while in the present investigation RT difference scores were used. There is much evidence suggesting that accuracy and RT could be related to different neural and cognitive processes (Santee and Egeth 1982; Kahana and Loftus 1999; van Ede et al. 2012). Therefore, a contributing factor to the discrepant result in the current study and previous research could be related to the use of different behavioral outcome measures.

The finding that brain iron concentration was positively linked to ISL in both samples of younger and older adults (Study 1) as well as a large study of older adults (Study 2) opens up for the possibility that brain iron might be differentially associated with implicit and explicit learning. Although the interaction between declarative and non-declarative memory systems during ISL is not fully understood, one possibility is that brain iron may lead to functional suppression of explicit memory operations thereby facilitating ISL. This line of reasoning is in agreement with the idea of competition between declarative and non-declarative memory systems (Poldrack and Packard 2003), and with results showing that disruption of declarative memory may actually facilitate motor skill learning (Brown and Robertson 2007). Also, some studies have shown that weaker frontal-lobe-related functions can result in enhanced implicit learning (Filoteo et al. 2010; Galea et al. 2010; Virag et al. 2015), indicating an inverse relationship between ISL and executive functions. These results suggest a competition between different, but potentially overlapping, fronto-striatal networks underlying these functions (Poldrack et al. 2001; Poldrack and Packard 2003). Thus, higher striatal iron concentration may disrupt competing explicit cognitive processes, resulting in more efficient ISL. Our results thus stress the need to consider the type of task used and the cognitive system under study as moderating factors in the relationship between brain iron and behavior. Moreover, an important question for future studies would be to examine the extent to which BG iron concentration specifically affects ISL, or if it also influences other forms of implicit learning. Another important question relates to whether BG iron concentration affects only online ISL or if the effect also transfers to offline consolidation of implicit learning.

The association among striatal iron accumulation, task-related brain activation, and ISL should be interpreted with some limitations in mind. First, Study 1 included a limited number of participants, and future studies with larger sample sizes are needed to confirm the link between BG iron concentration and brain activation related to ISL. Moreover, the current samples of older adults most likely consisted of high-performing individuals, and may, therefore, not be representative of the general older population. Second, Study 2 used metrics derived from functional images to estimate brain iron concentration, which lack sensitivity compared to QSM. While we acknowledge that V_EPI_ estimates of iron concentration is less sensitive and specific to brain iron compared to gradient-echo sequences, it may serve the purpose of further exploring data acquired using more precise methods. Moreover, as associations between brain iron and ISL converged between the two studies using different techniques, it is unlikely that the metric used in Study 2 is completely insensitive to brain iron concentration. Third, it has been proposed that individual differences in implicit learning are not stable over time and that many ISL tasks, including the SRT, have low reliability (Stark-Inbar et al. 2017). Although the current study did not permit calculating test–retest reliability measures, a recent study (Kalra et al. 2019) suggested that while SRT reliability was lower than for episodic memory, learning measures correlated across testing sessions indicating that implicit learning in test session 1 was indeed predictive of learning in test session 2 on an individual basis. In future studies, the use of the alternate SRT (Howard and Howard 1997) should be considered, since this task has demonstrated better reliability while minimizing the use of explicit strategies (Howard and Howard 1997; Janacsek et al. 2012; Stark-Inbar et al. 2017).

We examined the relationship between brain iron concentration and biobehavioral measures of ISL. There were three main findings. First, BG iron concentration was positively related to SL in two independent studies. Second, ISL was robust for both younger and older adults, and performance-related activation was found in a fronto-striatal network across both age groups. Third, BG brain iron concentration was positively linked to task-related BOLD signal in frontal and subcortical regions. These results indicate that among healthy younger and older individuals, brain iron might facilitate implicit motor learning.
